# Evaluating large language models on a highly-specialized topic, radiation oncology physics

**DOI:** 10.3389/fonc.2023.1219326

**Published:** 2023-07-17

**Authors:** Jason Holmes, Zhengliang Liu, Lian Zhang, Yuzhen Ding, Terence T. Sio, Lisa A. McGee, Jonathan B. Ashman, Xiang Li, Tianming Liu, Jiajian Shen, Wei Liu

**Affiliations:** ^1^ Department of Radiation Oncology, Mayo Clinic, Phoenix, AZ, United States; ^2^ School of Computing, The University of Georgia, Athens, GA, United States; ^3^ Department of Radiology, Massachusetts General Hospital, Boston, MA, United States

**Keywords:** large language model, natural language processing, medical physics, artificial intelligence, ChatGPT

## Abstract

**Purpose:**

We present the first study to investigate Large Language Models (LLMs) in answering radiation oncology physics questions. Because popular exams like AP Physics, LSAT, and GRE have large test-taker populations and ample test preparation resources in circulation, they may not allow for accurately assessing the true potential of LLMs. This paper proposes evaluating LLMs on a highly-specialized topic, radiation oncology physics, which may be more pertinent to scientific and medical communities in addition to being a valuable benchmark of LLMs.

**Methods:**

We developed an exam consisting of 100 radiation oncology physics questions based on our expertise. Four LLMs, ChatGPT (GPT-3.5), ChatGPT (GPT-4), Bard (LaMDA), and BLOOMZ, were evaluated against medical physicists and non-experts. The performance of ChatGPT (GPT-4) was further explored by being asked to explain first, then answer. The deductive reasoning capability of ChatGPT (GPT-4) was evaluated using a novel approach (substituting the correct answer with “None of the above choices is the correct answer.”). A majority vote analysis was used to approximate how well each group could score when working together.

**Results:**

ChatGPT GPT-4 outperformed all other LLMs and medical physicists, on average, with improved accuracy when prompted to explain before answering. ChatGPT (GPT-3.5 and GPT-4) showed a high level of consistency in its answer choices across a number of trials, whether correct or incorrect, a characteristic that was not observed in the human test groups or Bard (LaMDA). In evaluating deductive reasoning ability, ChatGPT (GPT-4) demonstrated surprising accuracy, suggesting the potential presence of an emergent ability. Finally, although ChatGPT (GPT-4) performed well overall, its intrinsic properties did not allow for further improvement when scoring based on a majority vote across trials. In contrast, a team of medical physicists were able to greatly outperform ChatGPT (GPT-4) using a majority vote.

**Conclusion:**

This study suggests a great potential for LLMs to work alongside radiation oncology experts as highly knowledgeable assistants.

## 1 Introduction

The advent of large language models (LLM) has completely transformed natural language processing (NLP) (1). The traditional paradigm of NLP follows the typical pipeline of creating customized solutions for downstream applications through supervised training. For example, a pre-trained BERT (2) model must be modified with additional network layers and then fine-tuned on labeled training data to perform tasks such as sequence classification or question answering. In some situations, it might also be beneficial or necessary to further pre-train such models on domain specific data to attain acceptable performance (3, 4). For example, AgriBERT (5) was pre-trained on agriculture-related text data, to properly address NLP tasks in the food and agriculture domain. However, the expansive size and exceptional few-shot learning capabilities enable LLMs to solve NLP problems through *in-context learning*, which reduces or even eliminates the need for annotated training samples (6, 7). During in-context learning, LLMs generalize from a few examples (or no examples at all) based on *prompts*, which typically are descriptive user inputs that characterize desired responses from LLMs (6, 8). For example, “summarize the following text” is a straightforward prompt that asks the language model to produce a summary for the input text. In general, LLMs provides a novel and simplified workflow for NLP that could potentially do away with supervised fine-tuning and its associated intricacies such as hyper-parameter tuning and model architecture modification. Furthermore, in-context learning significantly reduces the need for expensive and time-consuming human annotation (6, 9). It is especially desirable in medicine and science due to the limited data available in these domains (4, 10–12).

In recent months, the world has witnessed the rise of ChatGPT ^1^, which has enjoyed significant global popularity given its unprecedented language capabilities and accessibility to the general public through a chatbot interface. ChatGPT is based on the powerful GPT-3 model (6), one of the first large language models in history. The 175-billion-parameters GPT-3 was trained on a large data collection that encapsulated diverse Internet data (including the Common Crawl ^2^ and Wikipedia ^3^). It demonstrates exceptional performance in a variety of NLP tasks spanning from text summarization to named entity recognition (NER) through its text generation objective (indeed, many NLP tasks can be translated to some forms of text generation). ChatGPT inherits these capabilities from GPT-3, along with the massive knowledge on diverse topics stored in the parameter space. More importantly, ChatGPT was trained through *Reinforcement Learning from Human Feedback* (RLHF), a reinforcement learning process that incorporates human preferences and human ranked values through user feedback. This process tunes the model to generate outputs that are most appealing and relevant to human users. The capabilities of ChatGPT empowers diverse practical applications ranging from essay writing to code generation (13).

One of the most powerful LLM to date is GPT-4 ^4^, a successor to GPT-3. While OpenAI has not revealed much about its technical details yet, GPT-4 has demonstrated superior performance over the GPT-3.5-based ChatGPT in various scenarios (9, 12, 14, 15). In fact, as of March 2023, GPT-4 is powering Microsoft’s search engine, Bing (16), which demonstrates the potential of LLM-based search. In addition, unlike its predecessors, GPT-4 is a multi-modal model that accepts image inputs, which undoubtedly leads to more interesting applications.

GPT-4 has been shown to perform exceptionally well on various academic and professional benchmarks (14). For example, GPT-4 passes the USMLE exam with a >20% margin (17). In fact, GPT-4 scores at over the 90th percentile on the SAT, the Uniform Bar Exam and the verbal section of the GRE (see Figure 4 in the “GPT-4 Technical Report” (14)), where almost all of them included a multiple-choice component. Indeed, multiple-choice examinations are common for evaluating LLMs (14, 18, 19). Most multiple-choice exams that have been used to evaluate LLMs are based on topics that are among the most well represented in academics. For example, in 2022, the AP physics exam had 144,526 test-takers (20), the LSAT had 128,893 test-takers (21), the GRE had approximately 342,000 test-takers (22). As a result of the large numbers of test-takers taking these exams as well as the importance placed on scores in determining university admittance, there exists an exceeding amount of resources (including text data accessible on the internet). Regardless of the particular LLM under evaluation, the ease of access and overall ubiquity of these tests and relevant test preparation materials effectively preclude a high performance when evaluating LLMs on these tests. It is therefore important to also study LLMs on more obscure and specialized topics where the size of the training data is likely much smaller. In 2022, there were only 162 medical school graduates, who applied for radiation oncology residency programs (23). Radiation oncology physics therefore represents a topic that is relatively unknown to the general population and may therefore be a more fair test in evaluating LLMs as compared to highly represented knowledge-bases. Obscure topics may represent the greatest educational opportunity and also the greatest risk for the general population in the context of LLMs, as the responses may be more relied upon while being less accurate and with mistakes being less likely to be noticed.

An important factor in evaluating the accuracy of LLMs is to ensure that the test questions are left out of the training data (24), i.e. not contaminated. The best way to ensure this is to create new questions for testing. In this study, a multiple-choice examination has been created for this purpose. Four transformer-based LLMs have been chosen for evaluation: ChatGPT (GPT-3.5) (6), ChatGPT (GPT-4) (14), Bard (LaMDA) (25), and BLOOMZ (26). These results are compared to radiation oncology experts as well as non-experts. Additionally, ChatGPT (GPT-4) is further explored on how to improve its answers and on its deductive reasoning capabilities. Experimental results indicate that GPT-4 attains the best performance among LLMs and outperforms professional medical physicists on average, especially when prompted to explain its reasoning before answering the question. We also conduct extensive ablation studies and analyses to comprehensively measure and explain the results.

## 2 Related work

### 2.1 Large language models

Transformer-based pre-trained language models (PLMs), such as BERT (2) and GPT (27), have revolutionized natural language processing. Surpassing previous methods (e.g., RNN-based models) in numerous tasks, they have promoted interest in and accessibility of language models (28). Generally, PLMs can be categorized into three types: autoregressive models (like GPT), masked language models (such as BERT), and encoder-decoder models (e.g., BART (29) and T5 (30)). More recently, there is a rise of very large language models, including GPT-3 (6), Bloom (31), PaLM (32), and OPT (33). Rooted in the transformer architecture, these models draw inspiration from the likes of BERT and GPT but are developed at much larger scales.

The objective of large language models is to accurately learn contextual and domain-specific latent feature representations from input text (28). For example, the vector representation of “discharge” might vary considerably between medical and general domains. Smaller language programs often require continual pre-training and supervised fine-tuning on downstream tasks to achieve acceptable performance (3, 4). However, very large language models could potentially eliminate the need for fine-tuning while maintaining competitive results (6).

Besides the progress in model architecture, scale and training strategies, large language models can be further aligned with human preferences through reinforcement learning from human feedback (RLHF) (34). This approach has been implemented in various LLMs, such as Sparrow (35), InstructGPT (36), and ChatGPT. InstructGPT was based on GPT-3 and was trained through a process during which user preferences were prioritized through human-generated ranking feedback. As a successor to InstructGPT, ChatGPT also employs RLHF, focusing on adhering to prompts and generating comprehensive responses. OpenAI also implemented guardrails to prevent the generation of biased and undesirable outputs (31). ChatGPT has become a highly successful AI chatbot, capitalizing on GPT-3.5’s capabilities to facilitate human-like interactions.

RLHF incorporates human feedback into the generation and selection of optimal results by training a reward model based on human annotators’ rankings of generated outcomes (37). This reward model then rewards outputs that best correspond to human preferences and values. We believe these groundbreaking innovations make ChatGPT the perfect candidate for this study.

The recent development of GPT-4 has significantly advanced the state-of-the-art of language models. GPT-4 demonstrates enhanced reasoning abilities, creativity, image comprehension, context understanding, and multi-modal abilities, leading to more sophisticated and diverse responses. The success of large GPT models spurs exploration into specialized variants for specific fields, such as dedicated large language models for medical and healthcare applications, which could potentially revolutionize these domains.

### 2.2 Language models and examination

Large language models have exceptional natural language comprehension abilities. In addition, they are trained on massive data that supplies substantial knowledge. These characteristics make large language models ideal candidates for academic and professional benchmarks.

OpenAI recently released the first study in the literature that evaluates large language models on academic and professional exams designed for educated humans (14). The results indicate that GPT-4 performs extremely well on a wide variety of subjects ranging from the Uniform Bar Exam to GRE. In addition, a study from Microsoft indicates that GPT-4 can pass USMLE, the professional exam for medical residents, by a large margin (17).

This study is the first evaluation of large language models in the realms of radiation oncology and medical physics, and we believe it can inspire future research in evaluating LLMs on highly-specialized branches of medicine.

### 2.3 Prompt engineering

Collecting and labeling data for training or fine-tuning NLP models can be resource-intensive and costly, especially in the medical domain (4, 9, 12). Recent studies suggest that by employing prompts, large-scale pre-trained language models (PLMs) can be adapted to downstream tasks without the need for fine-tuning (6, 8).

A prompt consists of a set of instructions that customizes or refines the LLM’s response. Prompts extend beyond merely describing the task or specifying output formats. Indeed, they can be engineered to create novel interactions. For example, it is possible to prompt ChatGPT to emulate a cybersecurity breach with simulated terminal commands (38). In addition, prompts can also be used to generate additional prompts through a self-adaptation process (38).

The emergence of prompt engineering signifies the start of a new era in natural language processing (8). There is no doubt that carefully crafted prompts have much potential for diverse and sophisticated applications. However, determining the ideal prompt poses a unique challenge in the age of large language models. Currently, prompts can be designed manually or generated automatically (8, 39). Although automatically produced prompts may outperform manual prompts in certain tasks (8), they often suffer from poor human-readability and explainability (8, 40). Consequently, manual prompt generation may be favored in domains where interpretability is crucial, such as clinical practices and research. In this study, we design a suite of prompts and chain-of-thought prompts based on our experience in radiation oncology and medical physics and evaluate their impact on large language models.

## 3 Methods

A 100-question multiple-choice examination on radiation oncology physics was created for this study by an experienced medical physicist. This exam includes questions on the following topics: basic physics (12 questions), radiation measurements (10 questions), treatment planning (20 questions), imaging modalities and applications in radiotherapy (17 questions), brachytherapy (13 questions), advanced treatment planning and special procedures (16 questions), and safety, quality assurance (QA), and radiation protection (12 questions). The seven exam categories and the associated number of questions for each category follows the official study guide of American Board of Radiology (41). Of the 100 questions, 17 require numeric calculation (math-based). The exam questions are listed in the **Appendix, Section A**.

### 3.1 Comparison between LLM scores and human scores

The 100-question multiple-choice test on radiation oncology physics was inputted to each LLM in 5 separate trials (Trial 1 - Trial 5), except BLOOMZ, which was only tested in one trial. Each trial, beginning on a new thread or after reset, began with an initialization prompt notifying the LLM that it was about to be tested. Next, the LLM was prompted with instructions and 20 questions in batches until the exam was complete. In each trial, the instructions indicated to the LLM that it should only return the correct answer with no justification. The instructions were included in each batch since it was observed that the LLMs were less likely to follow the instructions otherwise. In cases where the LLM could not accept 20 questions at a time, batches of 10 questions were used instead (Bard). In cases where not all the answers were returned by the LLM, the next batch would include the non-answered question(s) as well as the entire next batch. These occurrences were rare. In each test trial, the global prompt and instructions prompt were phrased differently in order to account for response-noise due to prompt-noise. The initialization prompts and instructions prompts are given in **Table 1**.

**Table 1:** The LLM prompts used in each trial.

Trial	Initialization prompt	Instructions prompt
Trial 1	I am a radiation therapy researcher. My research group would like to study the answers given by ChatGPT on the topic of radiation oncology physics. I will now proceed to ask questions about radiation oncology physics.	Instructions: For each multiple choice question, provide the correct answer without any justification.
Trial 2	I want to evaluate your knowledge on radiation oncology physics by asking some multiple choice questions.	Please give only the question label and the letter for the correct answer.
Trial 3	Please answer the following practice questions as if you were a resident preparing for board certification exams.	Only give the correct answer in your response. Do not explain your answers.
Trial 4	We want to test your understanding of radiation oncology physics. For this reason, we have created some questions to ask you.	In your response, only report the question label and the corresponding answer.
Trial 5	I will ask you some multiple-choice questions.	Instructions: Only respond with the correct letter choice.

LLM test scores and their distributions were compared between each other as well as with scores from two human groups, medical physicists and non-experts. The medical physicists group included four experienced board-certified medical physicists, three medical physics residents, and two medical physics research fellows. The non-expert group included six individuals with advanced degrees in either electrical engineering, computer engineering, or computer science, but with no known prior experience or education on radiation oncology physics. Each human test-taker was allowed 3 hours to take the exam, closed book, also permitting the use of a basic calculator. In comparing the LLM scores and human scores, the mean scores, consistency in scores, and confidence in answers were evaluated.

To quantify accuracy, the average score was calculated for each LLM by averaging the scores from each trial. For the human test groups, individual scores were averaged over the whole group.

To quantify the overall consistency of scoring success, the standard deviation and average correlation between trials, defined as the average of the upper values of the Pearson correlation matrix between trials, were calculated. The average correlation indicates how consistent the correct scores were between trials where 1 is interpreted as the distribution being identical, 0 is equivalent to the distribution being purely random, and -1 is interpreted as the distribution being perfectly anti-correlated.

In order to quantify the degree of confidence in the answers given by the LLMs and human groups, the number of correct answers for each question were counted across all trials. For example, if each LLM answered the same question correctly 5 times, then the percentage of questions where all 5 answers were correct was incremented by 1% (since there are 100 questions). Additionally, the test results were compared to the expected distribution that would occur if the test-taker were guessing at random. The expected number of correct answers in 5 trials, when randomly guessing, is approximately 0.25×5 = 1.25 on average (98/100 questions have 4 choices, 2/100 have 5 choices). Using this value, the number of correct answer occurrences for each question can be estimated following the resultant Poisson distribution.

Finally, ChatGPT (GPT-3.5 and GPT-4) and Bard scores were compared to human scores where the scores were calculated based on majority vote.

### 3.2 Improving ChatGPT (GPT-4) accuracy - explain first, then answer

Due to the nature of transformer-based LLMs predicting the next word based on the prior context, it has been shown that the accuracy of responses can be improved if a sufficiently large LLM is prompted to develop the answer in a step-wise manner (24, 42, 43). ChatGPT (GPT-4) was evaluated using this strategy to see if its score could be improved by prompting it to explain first, then answer. The initialization prompt was the same as in Trial 1, however the instructions prompt for Trial 1 was changed to the following: “Instructions: For each multiple choice question, first give an explanation for the answer followed by the correct answer (letter choice).” These test results were then compared with the original non-justified test results.

### 3.3 Testing ChatGPT (GPT-4) on its deductive reasoning ability

In a multiple-choice question, an LLM will be most successful when the question and answer are often used in the same context. However, what happens if the correct answer has no shared context with the question, such as when the answer is “None of the above”? In this case, the LLM must deduce the correct answer by rejecting all the other answers, all of which likely share context with the question. This scenario would seem to be especially challenging for an LLM. To study the deductive reasoning ability of ChatGPT (GPT-4), each question of the 100-question multiple-choice exam was modified. Each correct answer was removed and replaced with “None of the above choices is the correct answer.” Such a context-reduction transformation cannot be used on a human since a human would notice the pattern. Because of this, there are likely to be no examples of this sort of transformation to be found for tests that were designed for humans and were subsequently used in the training data for LLMs. It is assumed, then, that an LLM would not notice this pattern. The modified exam was given to ChatGPT (GPT-4) using the Trial 1 prompts and was subsequently tested for improving accuracy by explaining first, then answering as described in Section 3.2.

## 4 Results

### 4.1 Comparison between LLM scores and human scores

**Figure 1:**
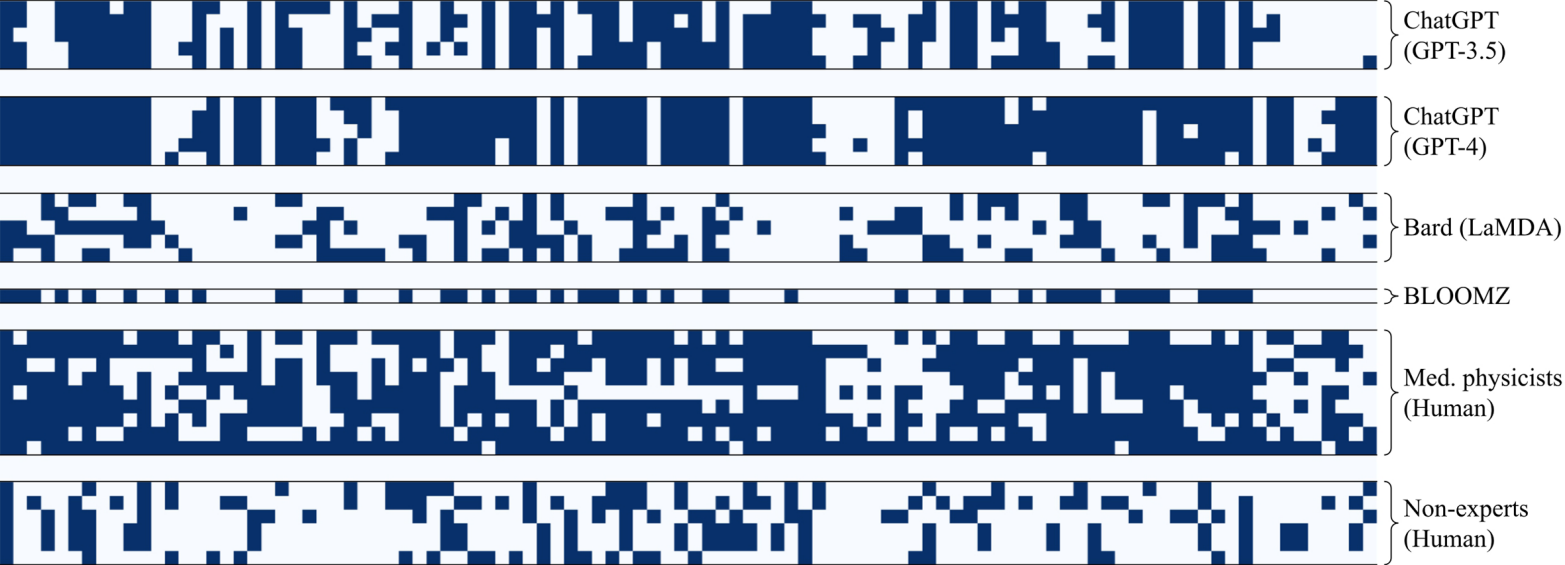
Raw marks for each test where the rows are separate tests and the columns are the test questions. Dark shaded squares represent correct answers.

**Figure 2:**
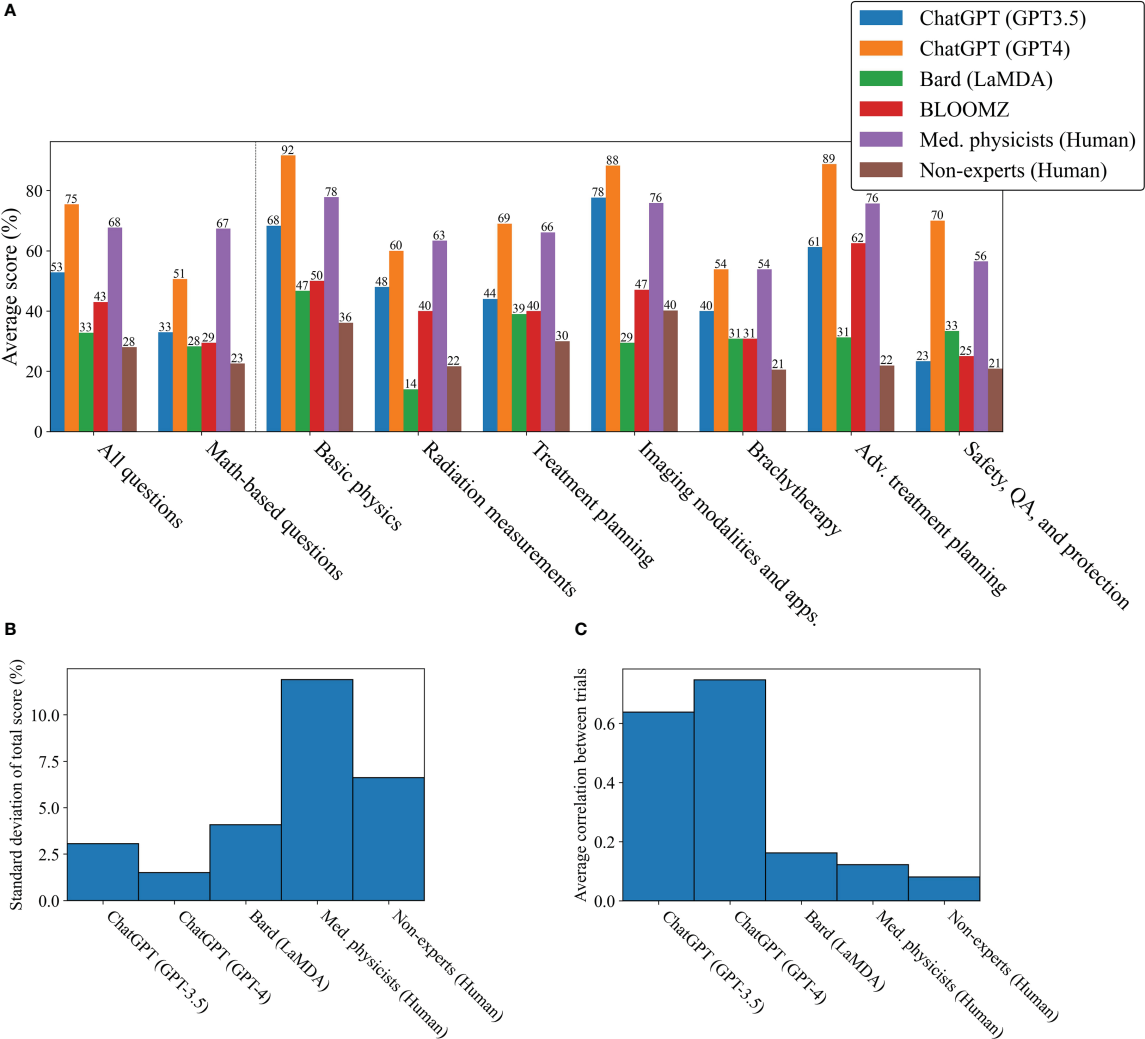
Overall performance and uncertainty in test results. **(A)** Mean test scores for each LLM by category. **(B)** Standard deviation in total scores. **(C)** Average correlation between trials.

The raw marks and mean test scores are shown in **Figures 1** and **2A** respectively, where the LLM mean test scores represent the mean of 5 trials (except for BLOOMZ - 1 trial) and the mean test scores for humans represent the mean of their respective groups (see Section 3.1). Each LLM was able to outperform the non-expert human group overall while only ChatGPT (GPT-4) outperformed the medical physicist group. For math-based questions, the medical physicists outperformed ChatGPT (GPT-4).

As can be observed in the raw marks shown in **Figure 1**, each LLM and human group showed variability between trials, not only in terms of uncertainty in the overall score, but also in terms of the number of times each question was answered correctly. The standard deviation and average correlation between trials are reported in **Figures 2B, C**. The LLMs were much more consistent in their scores and answers as compared to the human groups, showing both a low standard deviation in scoring and a high average correlation between trials.

From the results shown in 3, Bard slightly outperformed the non-expert group, however both groups performed similarly to a random guesser. ChatGPT (GPT-3.5 and GPT-4) and the medical physicists showed no similarity to random guessing. ChatGPT (GPT-3.5) was either confident, getting 35% of answers correct in each trial, or confused, getting 28% of answers incorrect. ChatGPT (GPT-4) was even more confident, getting 67% of questions correct in each trial, however it also showed a propensity for confusion, getting 14% of questions incorrect in each trial. As a group, the medical physicists were neither extremely confident, nor confused, however tending towards agreement in selecting the correct answers.

**Figure 3:**
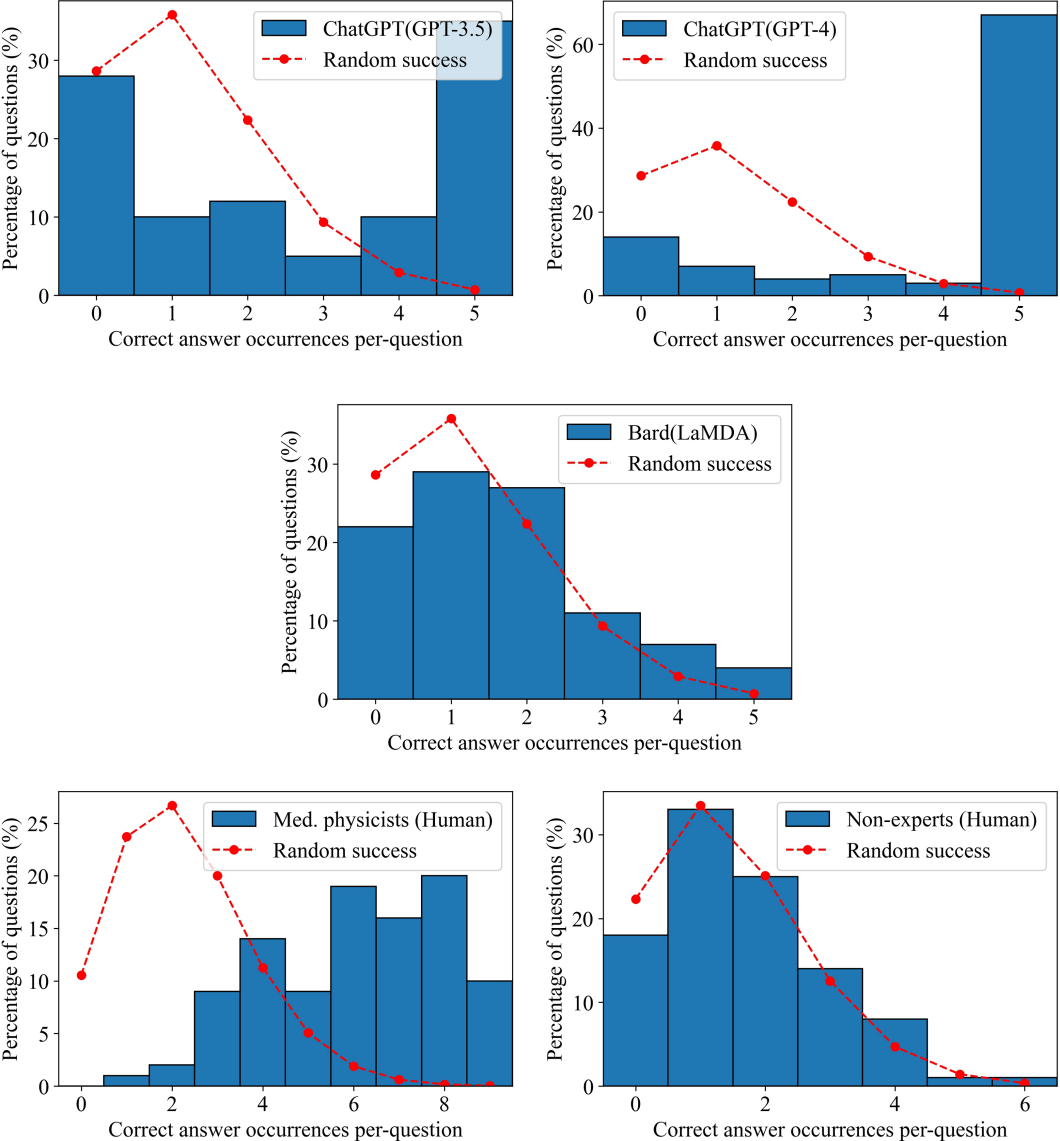
Confidence in answers. The number of correct answer occurrences per-question for each LLM and human group. The dashed red curve indicates the expected distribution if the answers were randomly selected based on the Poisson distribution.

**Figure 4:**
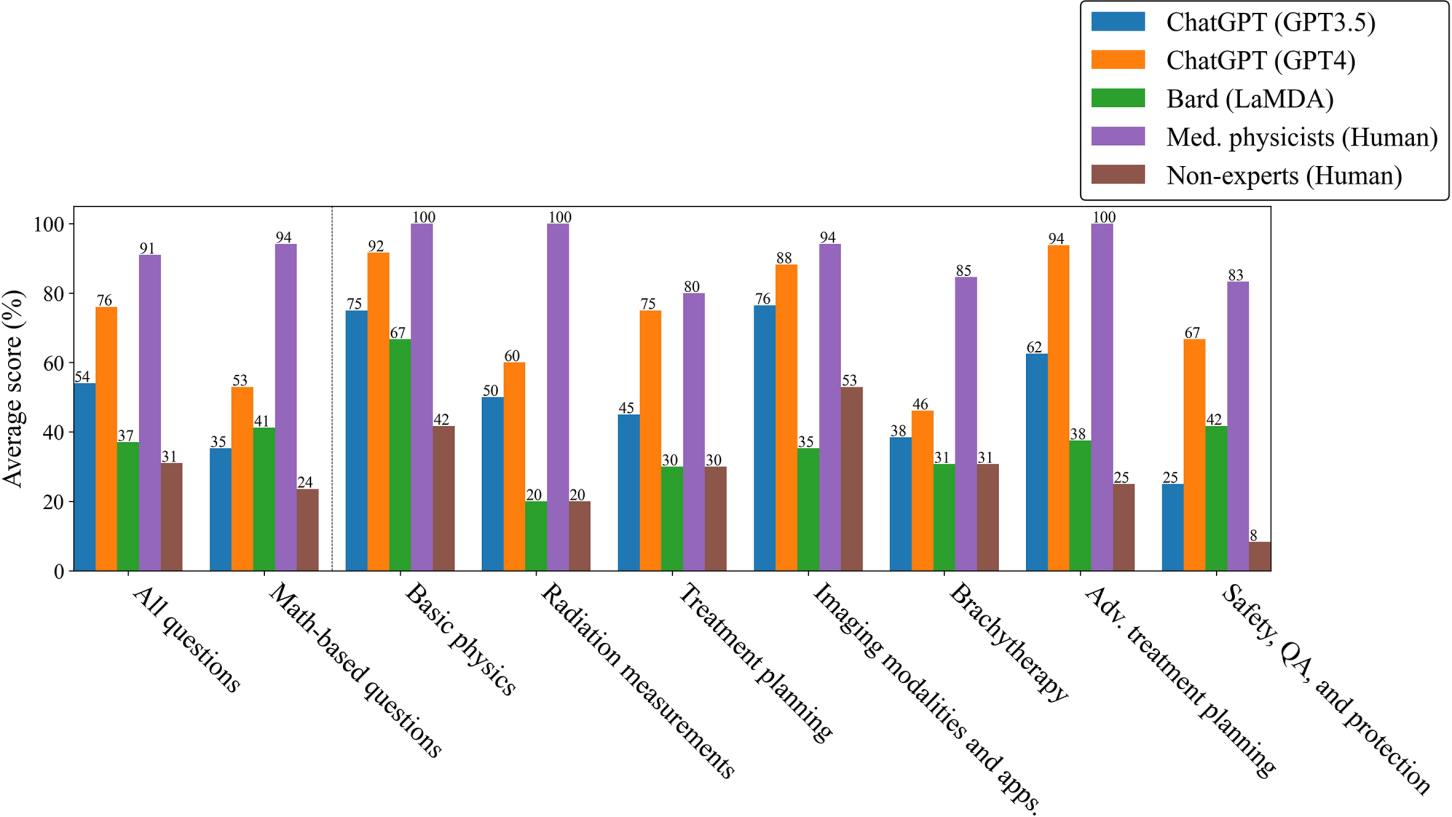
Scores by category, tabulated by majority vote among trials for LLMs and within the group for humans.

Although ChatGPT (GPT-3.5 and GPT-4) scored well overall, their scoring distributions, shown in **Figure 3**, suggested that if the LLMs could work together, there would be very little improvement in scoring, since they tended to be either confident or confused with low variability. Bard (LaMDA) and the non-expert groups would also likely show little improvement in working together as their answers tended towards random success. However, because medical physicists tended towards agreement on correct answers, it would be expected that their score would improve considerably when working together. To test for this, the answers for each group were combined using a “majority vote”. For each question, the most common answer choice was chosen as the group answer. In the case of a tie, one answer among the most common answer choices was chosen randomly. **Figure 4** shows the scoring results when utilizing a majority vote. As shown, ChatGPT (GPT-3.5 and GPT-4) improved very slightly, 1%. Bard (LaMDA) and the non-expert group improved by 4% and 3% respectively. However, the medical physicist group improved greatly, by 23%.

### 4.2 Improving ChatGPT (GPT-4) accuracy - explain first, then answer

**Figure 5:**
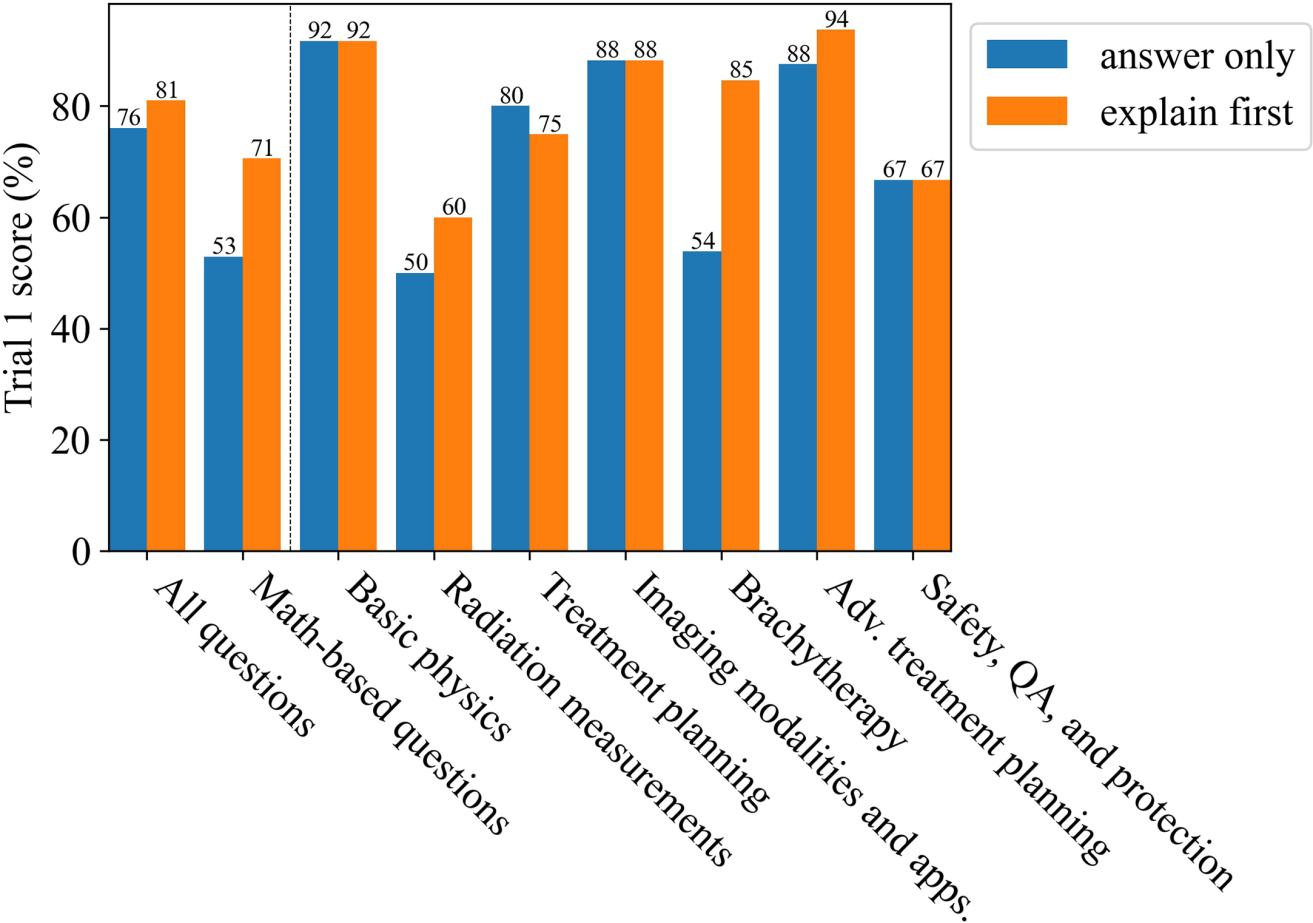
The improvement for Trial 1 as due to using the explain first, then answer method.

**Figure 5** shows the results for having prompted ChatGPT (GPT-4) to explain first, then answer, therefore allowing the answer to develop. ChatGPT’s (GPT-4) overall score improved by 5%, exceeding each prior trial. The greatest improvement was in the brachytherapy and math-based questions categories. These results are in agreement with prior studies that found this capability to be an emergent characteristic for sufficiently large LLMs (43). Sample responses from ChatGPT (GPT-4) are given in the **Appendix, Section A**.

### 4.3 Testing ChatGPT (GPT-4) on its deductive reasoning ability

**Figure 6:**
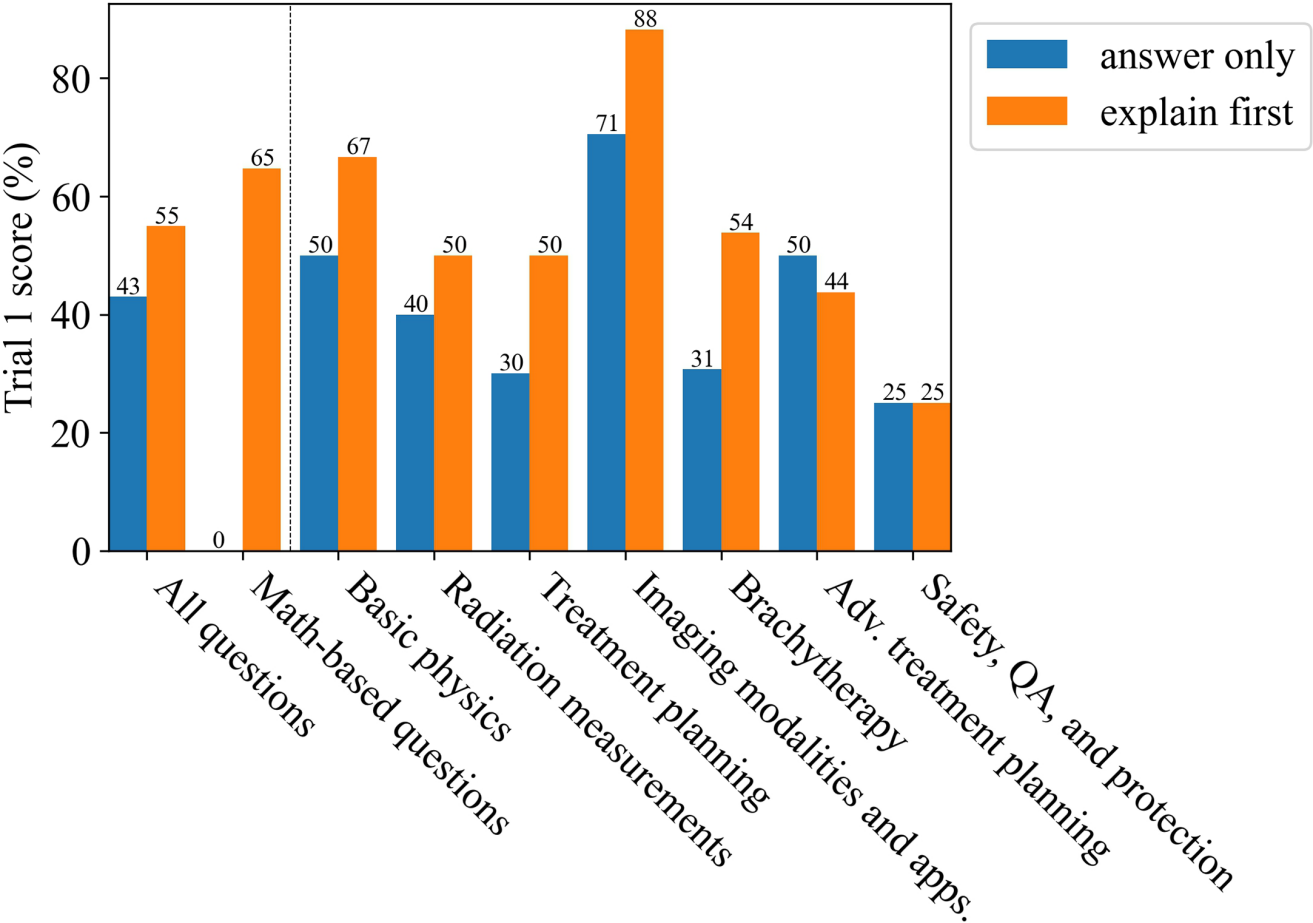
The scores for Trial 1 after replacing the correct answer with “None of the above choices is the correct answer. ”, a method for testing for deductive reasoning, and subsequent improvement as due to using the explain first, then answer method.

**Figure 6** shows the results for the deductive reasoning test where the correct answer was replaced by “None of the above choices is the correct answer” in all 100 questions. Overall, ChatGPT (GPT-4) performed much more poorly as compared to the original test. Although the performance was generally worse, the explain first, then answer method was especially important in improving its ability to deductively reason through the questions. Without explaining first, ChatGPT (GPT-4) got 0% of math-based questions correct, which improved to 65% after incorporating the explain first, then answer method, only one question less accurate than the original trial also using the explain first, then answer method.

## 5 Discussion

More than 1 million new cancer cases are diagnosed and more than 600,000 people die from cancer in the US every year. Radiotherapy (RT) is a standard treatment option used for nearly 50% of cancer patients (44–47). Physics plays an important role in radiation oncology due to the complexity and sophistication of physics and engineering adopted in modern radiation therapy. Therefore, it is essential for the radiation oncology professionals to understand radiation oncology physics well to ensure the safety and accuracy of the radiation treatment of cancer patients. The aim of this study was to evaluate LLMs on a highly-specialized topic, radiation oncology physics, based on a 100-question multiple choice exam that was specifically designed for this study. The exam can be found in the **Appendix, Section A**. The scoring results from the non-expert group suggest that the general population knows very little about radiation oncology physics as their scores were similar to random guessing. Bard (LaMDA) slightly outperformed the non-experts while BLOOMZ and ChatGPT (GPT-3.5 and GPT-4) greatly outperformed the non-experts. Amazingly, GPT-4 was able to outperform the average medical physicist in nearly all subcategories and improved its answer accuracy when prompted to explain its reasoning before answering (**Figures 2A**, **5**). As a general principle for improving accuracy, users should consider prompting ChatGPT to explain first, then answer. ChatGPT (GPT-4) showed a surprising ability to deductively reason in answering all 100 questions where each correct answer was modified to be “None of the above choices is the correct answer.”, particularly when it was prompted to explain first, then answer, scoring 55% overall. This result is somewhat perplexing and could potentially be an emergent property. Emergent properties are known to occur as the number of parameters is increased in LLMs (43). This novel method may be a useful method in determining whether deductive reasoning improves with the number of parameters going forward.

While ChatGPT (GPT-4) outperformed medical physicists overall, this study has also provided evidence that individual LLMs cannot compete with a small number of medical physicists working together (**Figure 4**). The likely reason is that humans vary significantly in capabilities and knowledge from individual to individual, even when their professional backgrounds are similar. Additionally, while an answer in a multiple-choice question will either be correct or incorrect, the scoring count distributions shown in **Figure 3** indicated that the medical physicists were far less likely to be confused, which, when aggregated over the whole group of medical physicists, allowed them to select the correct answer at a much higher rate in a majority vote. When ChatGPT (GPT-3.5 and GPT-4) was wrong, it was confidently wrong (confused). Similarly, when it was correct, it was confidently correct. Our results indicated that humans with expertise on a highly-specialized topic knew when to guess, how to guess intelligently, and were less likely to be wrong in their reasoning, even when the correct answer was not chosen. This comparison may not be completely fair as it is possible that if the exact same human could be tested repeatedly in the same manner as ChatGPT (GPT-3.5 and GPT-4), they might also repeat answers and show a degree of confusion individually. That point is arguably irrelevant, however, as there are many experienced medical physicists and only few LLMs as capable as GPT-4. The high degree of consistency in correct and incorrect answers for ChatGPT (GPT-3.5 and GPT-4) may be a sign of over-fitting (or memorization) in regards to radiation oncology physics knowledge. Regardless, being that radiation oncology physics is a highly-specialized topic, the performance of ChatGPT (GPT-4) was extraordinary and will likely continue to improve in the near-future. Practically speaking, this study suggests a great potential for radiation oncology experts to work alongside ChatGPT (GPT-4), using it as a highly knowledgeable assistant.

A weakness in evaluating LLMs using exams such as the one presented in this study is that this exam is not representative of the detailed and nuanced daily clinical work being performed by medical physicists and radiation oncology specialists. The relative performance between LLMs and medical physicists on radiation oncology physics exams reported in this study may therefore misrepresent the degree of equivalency between LLMs and individual medical physicists. Furthermore, GPT-4’s high performance on this certification-like exam, covering a highly specialized topic, suggests a degree of superficiality in the knowledge being assessed. Otherwise, we would have to entertain the possibility of GPT-4 being competent enough to fulfill the role of a medical physicist, which seems highly improbable. The radiation oncology community, and possibly the wider medical community, may therefore need to reevaluate certification procedures, as the necessity for humans to invest significant effort in acquiring such superficial knowledge will diminish as LLMs continue to advance. With this in mind, LLMs could potentially be used as a test for superficiality. Perhaps a greater focus on knowledge not known by the LLM should be more greatly emphasized.

### 5.1 Applying large language models in radiation oncology

This study is a continuation of a line of research that applies state-of-the-art NLP methods to radiation oncology. For example, Rezayi et al. (11) trained BioBERT on a large corpus of radiation oncology literature and a curated and anonymized text dataset from a hospital to build ClinicalRadioBERT, a specialized language model for radiation oncology. Liao et al. (48) proposed a framework of directing the attention of transformer-based language models to more important input tokens that significantly affect classification decisions. This method is particularly important for few-shot learning with few annotated samples, which is a common challenge in radiation oncology where it is difficult to collect and curate large amounts of multi-institution patient data that match certain requirements due to the concern of patient privacy. On a related note, ChatGPT has demonstrated superior performance as an effective text data augmentation approach over state-of-the-art text data augmentation methods in terms of testing accuracy and distribution of the augmented samples (9), which can also be used to address the few-shot learning challenge.

In addition, LLMs can be employed for innovative applications such as data de-identification. For example, GPT-4 outperforms ChatGPT and other language model competitors in de-identifying clinical notes with a 99% accuracy (12). This is of extreme importance to radiation oncology and all medicine specialities in general, since it is often cumbersome to anonymize data for cross-institution clinical collaboration and academic research. Some other applications of language models include building domain-specific knowledge graphs for oncology (49) without manual annotation from clinicians or other domain experts.

### 5.2 Multi-modal models in radiation oncology

Multi-modal models are the future of language model (1) and are important in medical diagnosis (50). Some early LLM studies with multi-modal data include ChatCAD (51), a framework to integrate images and texts for computer-aided diagnosis. It supports various diagnosis networks such as those for lesion segmentation and report generation. In this framework, ChatGPT can be used to enhance the outputs of these networks.

GPT-4 supports multi-modal inputs such as images, which further unlocks the potential of large language models in radiation oncology. It is necessary to investigate future models and applications that integrate text, images, dosimetric data, and other modalities into the diagnosis and treatment pipelines. We believe such multi-modal models display inherent affinity to the human brain (1) and future LLM models for medicine can receive inspirations from advances in both neuroscience and NLP.

## Data availability statement

The original contributions presented in the study are included in the article/**Supplementary Material**. Further inquiries can be directed to the corresponding authors.

## Author contributions

JH, JS, and WL contributed to conception and design of the study and edited the manuscript. JS designed the 100-question exam, subsequently edited by JH and WL. ZL, XL, and TL advised on LLM concepts and contributed to the manuscript. LZ and YD advised on the concept and experimental design. JH guided the writing process, wrote initial draft, performed all data analysis. TS, LM, and JA advised on clinical concerns and approved the manuscript. All authors contributed to the article and approved the submitted version.

## Footnotes

1
https://openai.com/blog/chatgpt

2
http://commoncrawl.org/

3
https://www.wikipedia.org/

4
https://openai.com/research/gpt-4
