# Supplementary material for: Evaluating large language models on a highly-specialized topic, radiation oncology physics
Source: Front Oncol. 2023 Jul 17;13:1219326. doi: 10.3389/fonc.2023.1219326 (PMC10388568; doi:10.3389/fonc.2023.1219326)
Supplement: Supplementary file 1 [file Table_1.pdf]

Appendix

A 100-question multiple-choice exam on radiation oncology physics

Correct answers = C, A, B, A, B, D, B, A, C, A, D, A, D, C, A, A, E, C, C, D, D, C, A, A, D, C, B, D, C, A, D, D, A, B, C, A, D, C, A, C, D, C, A, C, D, B, A, B, C, B, B, D, C, A, C, A, C, B, C, C, A, D, B, D, A, C, B, B, A, D, C, D, C, C, A, B, D, C, A, B, C, D, C, A, A, B, C, A, D, C, B, C, A, C, A, A, C, D, C, B

1. Which of the following particles cannot be accelerated by an electric field?
   (a) Protons
   (b) Electrons
   (c) Neutrons
   (d) Positrons
2. The mass of Carbon-12 (^{12}C) is _____ than the sum of the masses of 6 protons and 6 neutrons.
   (a) slightly smaller
   (b) slightly larger
   (c) equal to
   (d) All of the above are possible.
3. The binding energies for tungsten's K, L, and M shells are 70, 11, and 2 keV, respectively. A 50 keV photon interacts with tungsten through the photoelectric effect. What are the possible energies of the emitted photoelectron?
   (a) a continuous spectrum from 2 keV to 70 keV
   (b) 39 keV and 48 keV
   (c) 9 keV, 59 keV and 68 keV
   (d) 9 keV
4. Which of the following statements is true regarding the x-ray spectrum of an x-ray tube?
   (a) The maximum photon energy is determined by the kVp.
   (b) The maximum photon energy is determined by the target material.
   (c) The characteristic photon energies are determined by the tube current.
   (d) The characteristic photon energies are determined by the filtration.
5. The half value layer (HVL) for an x-ray beam is 2 mm aluminum. What is the minimum thickness of aluminum required to reduce the x-ray exposure by 50%?
   (a) 1 mm
   (b) 2 mm
   (c) 3 mm

(d) 4 mm

6. Which of the following component provides the high-power RF fields to accelerate electrons in a LINAC?
   (a) Thyratron
   (b) Target
   (c) Electron gun
   (d) Magnetron
7. What is the purpose of the “tongue and groove” design of multileaf collimator (MLC)?
   (a) Reduce frictions between leaves.
   (b) Reduce leakage doses between leaves.
   (c) Increase speed of leaves.
   (d) Increase field sizes for treatments.
8. When a LINAC is used to treat patients in electron mode, which of the following hardware is not needed?
   (a) Target
   (b) Electron gun
   (c) Monitor chamber
   (d) Scattering foil
9. Compared to a flattened beam, the flattening filter free beam ______.
   (a) Has higher average energy.
   (b) Is more penetrating.
   (c) Has a higher dose rate.
   (d) Increases treatment time.
10. The mass attenuation coefficient has the highest dependence on the atomic number of the medium and the energy of the incoming photon energy for ______.
   (a) Photoelectric effect
   (b) Compton scattering
   (c) Pair production
   (d) Rayleigh scatter
11. Which of the following is not an interaction between photons and a medium?
   (a) Photoelectric effect
   (b) Compton scattering
   (c) Pair production

(d) Bremsstrahlung

12. When the energy of the scattered photon is the minimum in a Compton interaction, what is the angle of the emitted Compton electron relative to the direction of the initial photon?

    (a) $0°$

    (b) $45°$

    (c) $90°$

    (d) $180°$

13. What is the relationship between the mass attenuation coefficient μ/ρ, mass energy transfer coefficient (μ_{tr}/ρ), and the mass energy absorption coefficient (μ_{en}/ρ)?

    (a) $\mu_{\mathrm{tr}}/\rho \geq \mu_{\mathrm{en}}/\rho \geq \mu/\rho$

    (b) $\mu_{\mathrm{en}}/\rho \geq \mu_{\mathrm{tr}}/\rho \geq \mu/\rho$

    (c) $\mu/\rho \geq \mu_{\mathrm{en}}/\rho \geq \mu_{\mathrm{tr}}/\rho$

    (d) $\mu/\rho \geq \mu_{\mathrm{tr}}/\rho \geq \mu_{\mathrm{en}}/\rho$

14. How does the KERMA compare to the absorbed dose for a 15 MV photon beam at 10 cm depth in a water phantom?

    (a) The KERMA is greater than the absorbed dose.

    (b) The KERMA is equal to the absorbed dose.

    (c) The KERMA is less than the absorbed dose.

    (d) The relationship between KERMA and the absorbed dose can't be determined.

15. Which of the following beams will not produce a buildup effect in the percent depth dose curve?

    (a) X-rays from a 100 kVp x-ray tube.

    (b) Gamma-rays from ^{60}Co.

    (c) 6 MV x-rays from a LINAC.

    (d) 18 MV x-rays from a LINAC.

16. Which device below is the most suitable for measuring lateral beam profiles?

    (a) Diodes

    (b) Farmer chambers

    (c) Plane parallel ionization chambers

    (d) Geiger-Muller counter

17. Four exposed films have the same optical density of 1.25. If all four films are placed on top of each other, what is the fraction of light that can transmit through?

    (a) 20%

    (b) 1%

(c) 0.1%

(d) 0.01%

(e) 0.001%

18. Which of the following devices is not suitable for in-vivo dose measurements?

    (a) Diodes

    (b) Thermoluminescent dosimeters (TLDs)

    (c) Ionization chambers

    (d) Optically stimulated luminescence dosimeters (OSLDs)

19. According to TG-51, which of the following correction factors is not needed when converting the measured charge to the generated charge in the ionization chamber?

    (a) Temperature and pressure correction

    (b) Recombination correction

    (c) Scatter correction

    (d) Polarity correction

20. For a farmer chamber calibrated at the standard temperature and pressure, which is the PTP factor for a local temperature of 23°C and pressure of 720 mmHg?

    (a) 0.97

    (b) 0.99

    (c) 1.03

    (d) 1.06

21. How is the beam quality defined in TG-51?

    (a) Nominal beam energy

    (b) Tenth value layer

    (c) Average beam energy

    (d) %dd(10)x

22. According to TG-51, a LINAC should have dose calibration in water tank _____.

    (a) Every day

    (b) Every month

    (c) Every year

    (d) Every other year

23. Why is TMR always larger than PDD at depths larger than d_{max}?

    (a) PDD includes both beam attenuation and beam divergence.

    (b) PDD includes beam attenuation but does not include beam divergence.

(c) TMR includes both beam attenuation and beam divergence.

(d) TMR does not include beam attenuation but does include beam divergence.

24. Which of the following changes will result in a larger geometric penumbra?

(a) Increasing SSD.

(b) Increasing the source to collimator distance.

(c) Reducing the source size.

(d) Move the patient closer to the collimator.

25. The output of a 6 MV beam is 1 cGy/MU at SSD=100 cm, depth=1.5 cm and field size 10 cm x10 cm. What is the output at SSD=98.5 cm, depth=1.5 cm and field size 10 cm x10 cm?

(a) 0.985 cGy/MU

(b) 0.97 cGy/MU

(c) 1.015 cGy/MU

(d) 1.03 cGy/MU

26. For a single field SAD treatment, the output at dmax is 1.02 cGy and the TMR at depth 10 cm is 0.7. What is the MU needed to delivery 100 cGy at depth 10 cm?

(a) 100

(b) 102

(c) 140

(d) 160

27. What wedge angle will produce the most uniform dose distribution for two treatment fields separated by 100°?

(a) 30°

(b) 40°

(c) 50°

(d) 60°

28. A patient's spine is treated by two adjacent fields with SSD=100 cm. The field lengths (symmetrical fields) in the superior-inferior directions are 20 cm and 24 cm, respectively. What is the gap in the skin to match the two spine fields at depth 10 cm?

(a) 1.0 cm

(b) 1.5 cm

(c) 2.0 cm

(d) 2.2 cm

29. For a craniospinal treatment treated with symmetrical fields, the field length for the upper spine field is 25 cm, and the field size for the lateral cranial fields are 20 cm x 20 cm. What is the collimator rotation angle for the cranial fields to align with the divergence of the spine fields?
    (a) 3°
    (b) 5°
    (c) 7°
    (d) 9°
30. The dose-volume-histogram (DVH) contains the following information except:
    (a) Location of the hot spot
    (b) Mean dose
    (c) Minimum dose
    (d) Maximum dose
31. What dose D5%=20 Gy to the heart on a DVH curve means?
    (a) 5% of the heart receives exactly 20 Gy.
    (b) 5% of the heart receives less than 20 Gy.
    (c) 95% of the heart receives more than 20 Gy.
    (d) 95% of the heart receives less than 20 Gy.
32. Which of the following parameters is sometimes changed by the dosimetrist during treatment planning?
    (a) Dose for each treatment fraction
    (b) Total treatment dose
    (c) Number of treatment fractions
    (d) Number of beams
33. For electron radiation therapy, which electron energy has the minimum PDD on the surface?
    (a) 6 MeV
    (b) 9 MeV
    (c) 12 MeV
    (d) 16 MeV
    (e) 20 MeV
34. Electron PDD has a low dose tail after the practical range. Which physical process is related to the generation of this tail?
    (a) Compton scattering
    (b) Bremsstrahlung radiation

(c) Characteristic x-rays

(d) Pair production

35. Which of the following treatments is suitable for SSD setup?

    (a) Four fields box for a pelvic treatment

    (b) Multiple fields IMRT treatment

    (c) Electron boost to chest wall of breast cancer

    (d) VMAT treatment with two arcs

36. Which of the following statements is true for electron effective SSD?

    (a) It increases with increasing electron energy.

    (b) It is equal to the distance from the x-ray sources to the isocenter.

    (c) It is larger than the distance from the x-ray sources to the isocenter.

    (d) It is independent of electron energy.

37. What is the range of a 6 MeV electron beam in the lung (0.25 g/cm^3)?

    (a) 3 cm

    (b) 6 cm

    (c) 9 cm

    (d) 12 cm

38. What is inverse planning in IMRT?

    (a) Choosing best beam angles.

    (b) Choosing appropriate beam modifiers such as wedges and compensators.

    (c) Optimizing beam fluence based on the desired dose constraints.

    (d) Adding beam block to protect organs at risk.

39. What is the purpose of the leaf sequencing algorithm in IMRT planning?

    (a) It converts optimized fluence to the achievable fluence by MLC.

    (b) It converts cost functions to the optimized fluence.

    (c) It converts achievable fluence to dose.

    (d) It converts desirable constraints to cost functions.

40. The dose gradient achievable in an IMRT plan is approximately ______.

    (a) 0.1%/mm

    (b) 1%/mm

    (c) 10%/mm

    (d) 50%/mm

41. Which of the following devices is not appropriate for IMRT patient QA?
    (a) Electronic portal imaging device
    (b) Film and ionization chamber
    (c) Diode Array
    (d) Single ionization chamber
42. Which parameter below is not typically set by a dosimetrist during IMRT treatment planning?
    (a) Beam energy
    (b) Beam angle
    (c) Beam weight
    (d) Upper and lower dose constraints
43. For material with HU=10, what is the ratio of linear attenuation coefficients between this material to water?
    (a) 1.01
    (b) 1.1
    (c) 0.99
    (d) 0.9
44. Which of the following methods can improve the spatial resolution of CT?
    (a) Increasing tube voltage.
    (b) Increasing field of view.
    (c) Decreasing field of view.
    (d) Decreasing tube current.
45. Why can't MR images replace CT for accurate dose calculations in radiation therapy?
    (a) MR images have inferior soft tissue contrast than CT.
    (b) MR images have worse spatial resolution than CT.
    (c) MR images deliver more ionization doses to the patient than CT.
    (d) MR images do not have information about electron density.
46. The velocity of ultrasound in soft tissue is 1500 m/s. The time to receive a signal after sending a pulse is 0.1 ms. What is the depth of the anatomical interface?
    (a) 5 cm
    (b) 7.5 cm
    (c) 10 cm
    (d) 15 cm
47. Which of the following imaging modalities utilizes non-ionizing radiation?

(a) MRI

(b) PET

(c) CT

(d) Radiograph

48. Which of the following statements is true about the spatial resolution of PET images?

    (a) PET images spatial resolution is better than CT and MRI.

    (b) PET images spatial resolution increases when the average energy of the emitted positrons decrease.

    (c) PET images spatial resolution increases when the number of detectors in the ring decreases.

    (d) PET images spatial resolution is better than 1 mm.

49. Which information below is not stored in a DICOM RT plan file?

    (a) Patient name

    (b) Treatment angles

    (c) CT scanner parameters, such as kVp, mAs

    (d) Prescription dose

50. A 4D CT has 10 phases, 200 slices in each phase. Each slice has 512x512 pixels, where each pixel uses 2 bytes. How long does it take to transfer this 4D CT over the internet with a transfer speed of 2 GB/second?

    (a) 0.2 s

    (b) 0.5 s

    (c) 1.0 s

    (d) 2.0 s

51. As compared to diagnostic CT, which statement below is not true for CT simulator?

    (a) CT simulator usually has a larger bore size than diagnostic CT.

    (b) CT simulator gives better image spatial resolution and contrast than diagnostic CT.

    (c) CT simulator needs extra laser for localizing the treatment isocenter.

    (d) CT simulator needs accurate geometry and HU for the whole body.

52. Which techniques below do not result in a reduced setup margin for planning target volume (PTV)?

    (a) Change weekly IGRT to daily IGRT.

    (b) Change the setup from laser to IGRT.

    (c) Add fiducial markers to the target.

    (d) Change 3D CRT plan to IMRT plan.

53. To get the best soft tissue contrast for a head and neck cancer treatment, which IGRT method is preferred?
    (a) MV portal imaging
    (b) kV orthogonal planar images
    (c) kV cone-beam CT
    (d) Surface imaging
54. Which of the following IGRT methods is not used for IMRT treatment?
    (a) MV portal image.
    (b) kV orthogonal planar images.
    (c) kV cone-beam CT.
    (d) CT on-rail.
55. Which IGRT method gives the lowest dose?
    (a) MV portal image
    (b) MV cone-beam CT
    (c) kV orthogonal planar images
    (d) kV cone-beam CT
56. Prostate motion can't be tracked by ______.
    (a) Infrared surface imaging.
    (b) Implanted RF beacons.
    (c) Implanted fiducial markers and fluoroscopy imaging.
    (d) MR images in MR-LINAC.
57. Which of the following is not an advantage of free-breathing gating for treating a lung tumor?
    (a) Reduced internal target volume.
    (b) Reduced dose to organ at risk.
    (c) Reduced treatment time.
    (d) Patient is comfortable in free breathing.
58. 4D CT is not needed for a ______.
    (a) tumor near the diaphragm
    (b) tumor in the brain
    (c) tumor in the lung
    (d) tumor in the liver
59. ______ is not a method for management of respiratory motion in radiation therapy.

(a) Deep-inspiration breath hold (DIBH).

(b) Real-time tumor tracking.

(c) IGRT using cone-beam CT.

(d) Free-breathing respiratory gating.

60. The half-life for a radioactive nuclide is 69 days. How much does it decay in one day?

(a) 0.5%

(b) 0.7%

(c) 1.0%

(d) 1.5%

61. Which statement is correct for an HDR source, ^{192}Ir, with activity 10 Ci?

(a) Its decay rate is equivalent to about 10 grams of ^{226}Ra.

(b) Its decay rate is equivalent to about 1 gram of ^{226}Ra.

(c) Its decay rate is 37 GBq.

(d) Its decay rate is 3.7 GBq.

62. The physical and effective half-lives for an unsealed isotope are 6 hours and 2 hours, respectively. What is the biological half-life?

(a) 12 hours

(b) 8 hours

(c) 4 hours

(d) 3 hours

63. What is the initial dose rate for a prostate ^{125}I seed implant to deliver a total dose of 120 Gy?

(a) 4.9 cGy/h

(b) 5.9 cGy/h

(c) 7.0 cGy/h

(d) 7.9 cGy/h

64. According to AAPM TG-43, ______ has the greatest anisotropy correction.

(a) ^{192}Ir

(b) ^{137}Cs

(c) ^{226}Ra

(d) ^{125}I

65. Which of the following is not a limitation of AAPM TG-43?

(a) The absorption and scattering in the source is not considered.

(b) The applicator is treated as water.

(c) The dose delivered while the source is in transit is usually ignored.

(d) The patient is treated as water.

66. Why is the radial dose function, g(r), approximately constant for an ^{192}Ir source for values of r less than 5 cm?

(a) There is no attenuation for the first 5 cm.

(b) Attenuation is included in the geometry factor.

(c) Attenuation is compensated by the increased scatter.

(d) Attenuation is included in the anisotropy factor.

67. An HDR vaginal cylinder case with a 3 cm diameter is planned to deliver 700 cGy to vaginal tissue at 5 mm from the cylinder surface. What is the approximate dose to the surface of the cylinder?

(a) 1244 cGy

(b) 933 cGy

(c) 700 cGy

(d) 500 cGy

68. To get a uniform dose at 5 mm from the cylinder surface in HDR vaginal treatment, the source dwell times ______.

(a) are the same at all dwell points.

(b) are longer at the ends.

(c) are longer in the middle.

(d) are longer at the superior end than the inferior end.

69. Why is the prescription dose for permanent prostate implant seeds lower when using ^{103}Pd as opposed to ^{125}I?

(a) The half-life for ^{103}Pd is shorter.

(b) The energy of emitted photon for ^{103}Pd is larger.

(c) The radial dose function g(r) for ^{103}Pd is larger.

(d) The anisotropy function for ^{103}Pd is larger.

70. ______ is not performed for HDR daily QA.

(a) Source position accuracy check

(b) Survey meter function check

(c) Radiation monitor function check

(d) Source calibration

71. ______ is the most appropriate device to find a lost radionuclide seed in the operation room.

(a) A well ionization chamber

(b) A Farmer chamber

(c) A Geiger-Muller counter

(d) A diode

72. Which of the following tests is not a typical procedure for HDR brachytherapy?

(a) Calibrating the source when a new source is used.

(b) Changing the source at a frequency of 3-4 months.

(c) Performing daily QA in the days only when patient treatment is scheduled.

(d) Performing patient surveys only before the treatment.

73. Which statement below is true for TBI treatment?

(a) Lung block is always needed when lateral opposed beams are used.

(b) FFF mode is used to increase the dose rate.

(c) The extended SSD increases the dose uniformity.

(d) The treatment is faster than it is for regular patients.

74. The maximum field size for a LINAC is 40 cm x 40 cm, and the height of a TBI patient is 180 cm. What is the minimum SSD required for the TBI treatment with a collimator angle of $0°$?

(a) 250 cm

(b) 350 cm

(c) 450 cm

(d) 550 cm

75. In total skin electron therapy (TSET), why is the gantry angle about $20°$ such that the central axis is above patient's head and below patient's feet?

(a) To minimize the dose from x-ray contaminations.

(b) To maximize the dose from x-ray contaminations.

(c) To increase the SSD.

(d) To reduce treatment time.

76. ______ is not an appropriate delivery method for intraoperative radiotherapy (IORT).

(a) MeV Electrons

(b) MV photons

(c) HDR Brachytherapy

(d) kV x-rays

77. Which of the following statements correctly describe the lateral penumbras of therapeutic proton beams?

(a) They are always larger than penumbras of MV x-rays in LINAC.

(b) They are always smaller than penumbras of MV x-rays in LINAC.

(c) They do not change with depth.

(d) They increase with depth.

78. ______ is not needed for a dedicated nozzle for proton pencil beam scanning.

(a) A dose monitor chamber

(b) Scanning magnets

(c) A range modulation wheel

(d) A spot position monitor

79. Which of the following statements is correct regarding range straggling in proton therapy?

(a) It is due to the statistical uncertainty in energy loss.

(b) It results in about 3.5% range uncertainty.

(c) It can be minimized by robust optimization.

(d) It is the same as range uncertainty.

80. Why do proton machines require a large gantry to bend protons, but LINACs don't?

(a) Protons at 250 MeV have a higher speed than electrons at 6-20 MeV.

(b) The mass of protons is about 1800 times more than the mass of electrons.

(c) Protons have positive charge while electrons have negative charge.

(d) Protons do not generate x-rays by Bremsstrahlung radiation.

81. Which of the following statements is incorrect regarding intensity-modulated proton therapy (IMPT)?

(a) An IMPT plan can be robust with robustness optimization.

(b) IMPT is more conformal than double scattering.

(c) IMPT is less sensitive to motion than double scattering.

(d) IMPT by pencil beam scanning needs less hardware than double scattering.

82. Which of the following statements about RBE for protons is correct?

(a) It is always a constant 1.1 for all depths.

(b) It is the highest at the shallow depth.

(c) It is the highest at the center of spread-out Bragg peak.

(d) It is the highest at the distal fall off depth.

83. Which device is not appropriate to measure the lateral profile of small fields in SRS?

(a) Diode

(b) Pin-point ionization chamber

(c) Farmer chamber

(d) Diamond detector

84. What is the purpose of using tertiary collimators in SRS technique?

(a) To reduce the penumbra.

(b) To reduce treatment time.

(c) To reduce collisions.

(d) To merge multiple isocenters to a single isocenter.

85. Which of the following statements is not true about SRS?

(a) A flattening filter free (FFF) can't be used in SRS technique.

(b) A Winston-Lutz test is required to verify the coincidence between radiation and mechanical isocenters.

(c) The SRS field may not have lateral charge particle equilibrium (CPE).

(d) Both frame-based and frame-less systems are feasible for SRS.

86. When planning for SRS/SBRT, which of the following statements is not typically a priority?

(a) Rapid dose fall off outside of target.

(b) Homogenous dose inside the target.

(c) Conformality index to be close to 1.

(d) Gradient index to be as small as possible.

87. Compared to regular fractionated treatment, _____ is not required for SRS technique.

(a) a smaller CT slice thickness

(b) a smaller dose calculation grid

(c) a higher output accuracy

(d) a higher precision in the coincidence of beam and gantry/couch isocenters.

88. Which SRS delivery technique can treat two brain metastases simultaneously using a single isocenter treatment plan.

(a) Arc plan with dynamic MLC.

(b) Arc plan with cones.

(c) Dynamic conformal Arc.

(d) Arc plan with static MLC.

89. _____ is a test for MLC leaf positions.

(a) Start shot for gantry isocentricity.

(b) Winston-Lutz test.

(c) Start shot for MLC isocentricity.

(d) Picket fence test.

90. According to TG-142, which test does not need to be performed daily?

(a) Door interlock

(b) Output

(c) Light field and radiation field coincidence

(d) Laser accuracy

91. Which of the following parameters is the most important for cone beam CT (CBCT) used in IGRT?

(a) HU accuracy

(b) Coincidence between the CBCT isocenter and the radiation beam isocenter

(c) Spatial resolution

(d) Field of view

92. According to TG-142, which is the tolerance for monthly output constancy?

(a) 0.5%

(b) 1%

(c) 2%

(d) 3%

93. The quality assurance test for ______ is not addressed in TG-142.

(a) CT simulator

(b) MLC

(c) CBCT

(d) Planar MV imager

94. Which of the following parameters is not needed in shielding calculations for the 2nd barriers?

(a) Distance.

(b) Occupancy factor.

(c) Use factor.

(d) Workload.

95. What is the occupancy factor used in shielding design for the nurse station next to a LINAC room?

(a) 1

(b) 1/5

(c) 1/20

(d) 1/40

96. The proper order of materials for LINAC treatment room doors (from inside to outside) is ______.
    (a) Steel, Borated polyethylene, Lead
    (b) Lead, Borated polyethylene, Steel
    (c) Borated polyethylene, Lead, Steel
    (d) Borated polyethylene, Steel, Lead
97. How many TVLs are needed to reduce the exposure rate to 1/5 of the original value?
    (a) 0.3
    (b) 0.5
    (c) 0.7
    (d) 1.0
98. ______ has the highest radiation weighting factor.
    (a) 6 MV x-rays
    (b) 250 MeV protons
    (c) 6 MeV electrons
    (d) 100 keV neutrons
99. Which of the following errors is not considered a Radiation Medical Event for a 5-fraction SBRT prostate treatment?
    (a) A total dose deviation of 25%.
    (b) The wrong patient was treated.
    (c) A single fraction dose deviation of 45%.
    (d) Dose was delivered to the liver.
100. Which of the following factors is not needed for a FMEA process map in CT simulation of lung cancer?
    (a) Documenting immobilization devices used in CT sim.
    (b) Verifying bore size of CT sim.
    (c) Verifying HU accuracy of CT Sim in daily QA.
    (d) Verifying correct patient before CT Sim.

B Explain first, then answer responses from ChatGPT (GPT-4)

In order to show how the explain first, then answer prompt works in ChatGPT (GPT-4), we selected 10 questions here for demonstration, starting from the 61st question of the 100-question found in the Appendix, Section A.

61. 1 Curie (Ci) is equal to 3.7×10^{10} Becquerels (Bq). Therefore, 10 Ci of ^{192}Ir is equivalent to 37×10^{10} Bq or 370 GBq.
Answer: C. Its decay rate is 37 GBq.

62. The equation for effective half-life (T_{eff}) is $\frac{1}{T_{\text{eff}}} = \frac{1}{T_{\text{phys}}} + \frac{1}{T_{\text{bio}}}$, where T_{phys} is the physical half-life and T_{bio} is the biological half-life. Solving for T_{bio} gives $T_{\text{bio}} = \left(\frac{1}{T_{\text{eff}}} - \frac{1}{T_{\text{phys}}}\right)^{-1} = \left(\frac{1}{2} - \frac{1}{6}\right)^{-1} = 3\,\text{hours}$.
Answer: D. 3 hours

63. The initial dose rate (D_0) can be calculated using the formula $D_0 = \frac{D_{\text{total}}}{(T \times \ln(2))}$, where D_{total} is the total dose and T is the half-life of the isotope. For ^{125}I, the half-life is approximately 60 days. $D_0 = \frac{120\,\text{Gy}}{(60\,\text{days} \times \ln(2))} \approx 5.9\,\text{cGy/h}$.
Answer: B. 5.9 cGy/h

64. According to AAPM TG-43, ^{125}I has the greatest anisotropy correction among the listed isotopes.
Answer: D. ^{125}I

65. AAPM TG-43 does consider the absorption and scattering in the source, so this statement is not a limitation of the protocol.
Answer: A. The absorption and scattering in the source is not considered.

66. The radial dose function, $g(r)$, is approximately constant for a ^{192}Ir source for values of r less than 5 cm because the attenuation is compensated by the increased scatter.
Answer: C. Attenuation is compensated by the increased scatter.

67. The dose rate at 5 mm (0.5 cm) from the cylinder surface is 700 cGy. The dose at the cylinder surface (r = 1.5 cm) can be estimated using the inverse square law: $\text{Dose}_{\text{surface}} = \text{Dose}_{5\text{mm}} \times \left(\frac{5\,\text{mm}}{15\,\text{mm}}\right)^2 \approx 700\,\text{cGy} \times \left(\frac{1}{9}\right) = 77.8\,\text{cGy}$.
Answer: D. 500 cGy (closest to the calculated value)

68. To get a uniform dose at 5 mm from the cylinder surface in HDR vaginal treatment, the source dwell times are longer at the ends of the cylinder.
Answer: B. are longer at the ends.

69. The prescription dose for permanent prostate implant seeds is lower when using ^{103}Pd as opposed to ^{125}I because the half-life for ^{103}Pd is shorter.
Answer: A. The half-life for ^{103}Pd is shorter.

70. Source calibration is not performed for HDR daily QA, as it is typically done less frequently (e.g., monthly or quarterly).
Answer: D. Source calibration.
